# Implementation of a Web-Based Communication System for Primary Care Providers and Cancer Specialists

**DOI:** 10.3390/curroncol30030269

**Published:** 2023-03-21

**Authors:** Bojana Petrovic, Jacqueline L. Bender, Clare Liddy, Amir Afkham, Sharon F. McGee, Scott C. Morgan, Roanne Segal, Mary Ann O’Brien, Jim A. Julian, Jonathan Sussman, Robin Urquhart, Margaret Fitch, Nancy D. Schneider, Eva Grunfeld

**Affiliations:** 1 Dalla Lana School of Public Health, University of Toronto, Toronto, ON M5T 3M7, Canada; 2 Department of Family and Community Medicine, Temerty Faculty of Medicine, University of Toronto, Toronto, ON M5G 1V7, Canada; 3 Cancer Rehabilitation and Survivorship, Department of Supportive Care, Princess Margaret Cancer Centre, Toronto, ON M5G 2C4, Canada; 4 Institute of Health Policy, Management and Evaluation, University of Toronto, Toronto, ON M5T 3M7, Canada; 5 Bruyère Research Institute, Ottawa, ON K1N 5C8, Canada; 6 Department of Family Medicine, Faculty of Medicine, University of Ottawa, Ottawa, ON K1G 5Z3, Canada; 7 Ontario Health East, Ottawa, ON K1J 1J8, Canada; 8 The Ottawa Hospital Cancer Centre, Ottawa, ON K1H 8L6, Canada; 9 Department of Radiology, Radiation Oncology and Medical Physics, Faculty of Medicine, University of Ottawa, Ottawa, ON K1N 6N5, Canada; 10 Department of Oncology, Faculty of Health Sciences, McMaster University, Hamilton, ON L8V 5C2, Canada; 11 Department of Community Health and Epidemiology, Faculty of Medicine, Dalhousie University, Halifax, NS B3H 1V7, Canada; 12 Department of Surgery, Nova Scotia Health, Halifax, NS B3H 2Y9, Canada; 13 Bloomberg Faculty of Nursing, University of Toronto, Toronto, ON M5T 1P8, Canada; 14 CanIMPACT Patient Advisory Committee, Toronto, ON M5G 1V7, Canada; 15 Ontario Institute for Cancer Research, Toronto, ON M5G 1N8, Canada

**Keywords:** electronic communication, coordination of care, cancer, primary care, implementation

## Abstract

Healthcare providers have reported challenges with coordinating care for patients with cancer. Digital technology tools have brought new possibilities for improving care coordination. A web- and text-based asynchronous system (eOncoNote) was implemented in Ottawa, Canada for cancer specialists and primary care providers (PCPs). This study aimed to examine PCPs’ experiences of implementing eOncoNote and how access to the system influenced communication between PCPs and cancer specialists. As part of a larger study, we collected and analyzed system usage data and administered an end-of-discussion survey to understand the perceived value of using eOncoNote. eOncoNote data were analyzed for 76 shared patients (33 patients receiving treatment and 43 patients in the survivorship phase). Thirty-nine percent of the PCPs responded to the cancer specialist’s initial eOncoNote message and nearly all of those sent only one message. Forty-five percent of the PCPs completed the survey. Most PCPs reported no additional benefits of using eOncoNote and emphasized the need for electronic medical record (EMR) integration. Over half of the PCPs indicated that eOncoNote could be a helpful service if they had questions about a patient. Future research should examine opportunities for EMR integration and whether additional interventions could support communication between PCPs and cancer specialists.

## 1. Introduction

Healthcare providers have reported challenges with addressing the needs of patients with complex health conditions, such as cancer, requiring the care of multiple healthcare providers. Often the first point of contact for medical care, primary care providers (PCPs) have reported challenges coordinating care with specialist physicians. In the 2019 Commonwealth Fund International Health Policy Survey of Primary Care Physicians, 58% of PCPs in Canada (where our study took place) reported that they are usually informed by specialists about changes to their patients’ medications or care plans [1]. In the same survey, Canada scored the second lowest among the 11 countries surveyed, with only 22% of PCPs indicating that they can electronically exchange patient summaries, 33% of PCPs indicating that they can electronically share laboratory and diagnostic test results, and 29% of PCPs indicating they can electronically share medications taken by their patient with physicians outside of their practice.

With the proliferation of digital technologies, there are new possibilities to improve healthcare delivery. Addressing complex health issues requires the sharing of information to enhance person-centered care [2]. As in other developed countries, electronic medical record (EMR) software has largely been implemented in Canada, with 93% of PCPs and 80% of specialists reporting EMR use [3]. However, EMRs have primarily been designed to support healthcare providers’ administrative tasks, such as billing and documentation [4]. Previous research has reported on the barriers to developing health information technology tools for care coordination, including: (1) lack of clarity about the meaning of coordination of patients with complex needs; (2) variation in clinical processes and healthcare provider roles; (3) difficulties with change management; and (4) uncertainty whether such tools and modified work patterns will improve healthcare providers’ compensation [5].

Managing patients with cancer requires collaboration among members of multi-disciplinary care teams [6], including the involvement of oncologists, oncology nurses, PCPs, and other healthcare providers [7]. Issues around the coordination of care for patients with cancer have been well documented, including poor communication between healthcare providers; lack of defined healthcare provider roles; physicians not being copied on patient reports; and incompatibility between different EMR systems [8,9]. These issues have led to the duplication of services, unnecessary appointments, and increased stress and confusion for patients with cancer [8,10,11].

Our multi-disciplinary team consisting of researchers, PCPs, cancer specialists, patients, and caregivers (Canadian Team to Improve Community-Based Cancer Care along the Continuum, CanIMPACT) conducted research aiming to identify gaps in care coordination for patients with cancer. Phase 1 research was followed by a stakeholder consultation to identify solutions to be tested in a Phase 2 intervention study. The selected solution (intervention), referred to as eOncoNote, was developed based on a recommendation to examine whether electronic consultation can facilitate cancer care coordination [12]. Within the WHO Classification of Digital Health Interventions [13], the eOncoNote system is an intervention aimed at healthcare providers, focusing on telemedicine (consultations for case management between healthcare providers) and healthcare provider communication. The eOncoNote system was designed to facilitate communication and coordination of care between PCPs and cancer specialists. The eOncoNote system was created as a cancer-specific modification of Champlain BASE eConsult [14], and was based on the same platform so that healthcare providers could use the same account to access both services. We studied the implementation of eOncoNote in the Ottawa region (Ontario, Canada), where the eConsult service has been available to PCPs since 2010 [14] and over half of the PCPs are registered users [15].

We report here the results of a sub-study that was part of a type 1 hybrid effectiveness-implementation trial. The sub-study was designed to answer two research questions: (1) what are PCPs’ experiences of implementing eOncoNote in their settings; and (2) how does access to eOncoNote influence communication between PCPs and cancer specialists?

## 2. Materials and Methods

### 2.1. Study Design and Intervention

The type 1 hybrid effectiveness-implementation trial design involves testing the effectiveness of an intervention while gathering data to examine the context of the intervention implementation [16]. In the larger study, the effectiveness aim was addressed through a pragmatic randomized controlled trial (pRCT), qualitative interviews with healthcare providers and patients, and a PCP survey. The implementation aim was addressed through qualitative interviews, analysis of eOncoNote system usage metrics, and data abstracted from the hospital EMR. Results from the pRCT [17] and qualitative interviews [18] have been reported separately. This paper focuses on the findings from the PCP survey and eOncoNote system usage data.

Cancer specialists and PCPs used the eOncoNote intervention in addition to usual methods of communication, such as telephone and fax. In contrast to the typical eConsult process which involves PCPs initializing communication with a specialist, eOncoNote discussions were initiated by the cancer specialist. While eConsult was designed to be a managed service where a PCP selects a specialty area to send a question rather than a specific healthcare provider, eOncoNote used the reverse process of having the oncology specialist initiate communication with the patient’s PCP. With both services, PCPs were able to download a PDF transcript of the discussion with a specialist, because eConsult and eOncoNote were not integrated with the PCP’s or specialist’s EMR software.

Once the patient was randomized to the eOncoNote intervention arm, the cancer specialist sent the PCP an invitation to communicate through the eOncoNote system. Whenever an eOncoNote message was sent, the recipient received an email alert that prompted them to log into the system to view the message and respond. If the PCP did not respond to the initial message, up to three reminders were sent to the PCP asking them to respond to the cancer specialist. Once the eOncoNote discussion was complete, PCPs were sent up to three prompts to complete the brief survey about their experience with eOncoNote and close the case.

Access to the eOncoNote system was free and available to cancer specialists and PCPs whose patients provided consent to participate. The duration of access to the eOncoNote system depended on whether the patient was in the treatment or survivorship phase, and their cancer disease site. For patients receiving treatment for prostate cancer or breast cancer, PCPs and cancer specialists used eOncoNote for 4 months or 6 months, respectively, to align with the duration of treatment. For patients with breast or colorectal cancer in the survivorship phase, PCPs and cancer specialists used eOncoNote for one year post-transfer to primary care.

### 2.2. Research Ethics

Approval was obtained from the Ottawa Health Science Network Research Ethics Board (#20170381-01H) and the Health Sciences Research Ethics Board at the University of Toronto (#34641) before starting patient recruitment.

### 2.3. Study Participants

Participants were PCPs and cancer specialists of patients who participated in the pRCT [17]. Patients were eligible to participate in the pRCT if they were receiving adjuvant chemotherapy for early-stage breast cancer, or radical or adjuvant radiation therapy for localized prostate cancer; or completed adjuvant therapy for breast or colorectal cancer with the intent of being discharged for survivorship follow-up care to their PCP. The PCPs of the participating patients were eligible to participate if: they were a licensed family physician or nurse practitioner; they were previously registered on Chaplain BASE eConsult; and their patient consented to be enrolled in the study (and they did not have any other patients enrolled, to control for possible contamination between intervention and control groups). Cancer specialists included medical oncologists and radiation oncologists, and nurses from the survivorship program.

### 2.4. Recruitment

Patients were recruited from a cancer center at an academic hospital in Ottawa, Ontario, Canada. The patient recruitment process has been described elsewhere [17]. Patient recruitment took place between February 2018 and March 2020.

### 2.5. Data Collection

#### 2.5.1. eOncoNote Data

Usage data were analyzed to report the extent of eOncoNote’s use for patients who were randomized to the intervention group. Usage data were analyzed from a communication log maintained by the research assistant (RA) and data on the eOncoNote system. The communication log contained dates when the RA prompted cancer specialists to send an eOncoNote message, and reminders to PCPs to respond to the cancer specialists and complete the survey. The log also tracked the type of PCP (i.e., nurse practitioner or family physician), as well as whether the PCP was previously registered on BASE eConsult. We tracked previous registration because we originally recruited patients whose PCPs may not have been registered on BASE eConsult. Shortly after beginning the pRCT, we modified the inclusion criteria to only include patients whose PCPs were previously registered on BASE eConsult, as we encountered challenges with contacting and registering PCPs. Usage data extracted from the eOncoNote system included information about the type of cancer specialist, dates when messages were sent, the number of messages sent, and the content of the eOncoNote messages.

#### 2.5.2. PCP Surveys

At the end of their eOncoNote discussion, PCPs whose patients were randomized to the intervention group were prompted to complete a brief 6-item survey within the system. The survey asked about PCPs’ perceived value of using eOncoNote. The brief PCP survey has been a standard part of the BASE eConsult system since this service was launched.

### 2.6. Data Analysis

Descriptive statistics were used to summarize the participant characteristics, eOncoNote usage, and PCP survey data. Summative content analysis [19] was used to summarize the topics of discussion between PCPs and cancer specialists. The summative approach involves identifying and quantifying content in the text [19].

## 3. Results

### 3.1. eOncoNote Data

eOncoNote communication log data were analyzed for 76 shared patients: 33 who were receiving active treatment and 43 who were in the survivorship phase. For seven patients, there were two discussions each within the system, including one patient in the treatment phase and six patients in the survivorship phase. The reasons for having two discussions per patient included: the original PCP was on leave and the second eOncoNote discussion invitation was sent to a locum (*n* = 1), there was an error in the original eOncoNote message sent from a cancer specialist (*n* = 3), and the PCP had an updated email address (*n* = 3).

Data regarding participant characteristics are presented in Table 1. There were seven oncologists and two nurses involved in eOncoNote discussions with PCPs. Since the nurses in the survivorship phase shared the patient caseload and were able to view each other’s eOncoNote discussions, both nurses were involved in discussions regarding 11 patients. Nearly all PCPs (74/76, 97%) were family physicians. Once the initial eOncoNote message was sent, over half of the PCPs (42/76, 55%) were sent three reminders to respond to the cancer specialist. Following the third and final reminder, 30 PCPs (30/76, 39%) responded to the cancer specialist within the eOncoNote system. In some instances, PCPs who did not respond within eOncoNote replied to the RA’s reminder message via email. Once the patient completed the pRCT and the cancer specialist sent the final message, over half of the PCPs (42/76, 55%) were sent three reminders to complete the brief survey and close the case.

Results from the eOncoNote case discussions are presented in Table 2. Most eOncoNote discussions were initiated within two weeks of the patient’s appointment with the cancer specialist. Cancer specialists initiated eOncoNote discussions promptly after being notified by the RA that the patient had been randomized to the intervention group (within 4 days for 82% of the cases in the treatment phase and 88% of the cases in the survivorship phase). Many of the PCPs (approximately 60% in each of the treatment and survivorship phases) did not respond to the eOncoNote invitation to communicate within the system. Once the patient completed their last questionnaire and the RA notified the cancer specialist to initiate case closure, 60% of the cancer specialists in the treatment phase and 79% of the cancer specialists in the survivorship phase initiated case closure within 9 days. Approximately half of the PCPs in the survivorship phase and about two-thirds of the PCPs in the treatment phase did not complete the survey to close the eOncoNote case discussion. Among the PCPs that responded to the eOncoNote invitation to communicate, nearly all (12/13, 92% in the treatment phase, and 15/17, 88% in the survivorship phase) sent only one message to the cancer specialist.

### 3.2. PCP Survey

Thirty-four PCPs completed the survey in eOncoNote, including 22 PCPs (22/34, 65%) who had a patient in the survivorship phase, seven (7/34, 21%) who had a patient who was receiving treatment for prostate cancer, and five (5/34, 15%) who had a patient who was receiving treatment for breast cancer. Nineteen PCPs (19/34, 56%) were based in Ottawa, and 15 PCPs (15/34, 44%) were based in other locations. Twenty-eight PCPs (28/34, 82%) practiced in an urban setting based on their postal code, while six (6/34, 18%) practiced in a rural setting.

**Figure 1:**
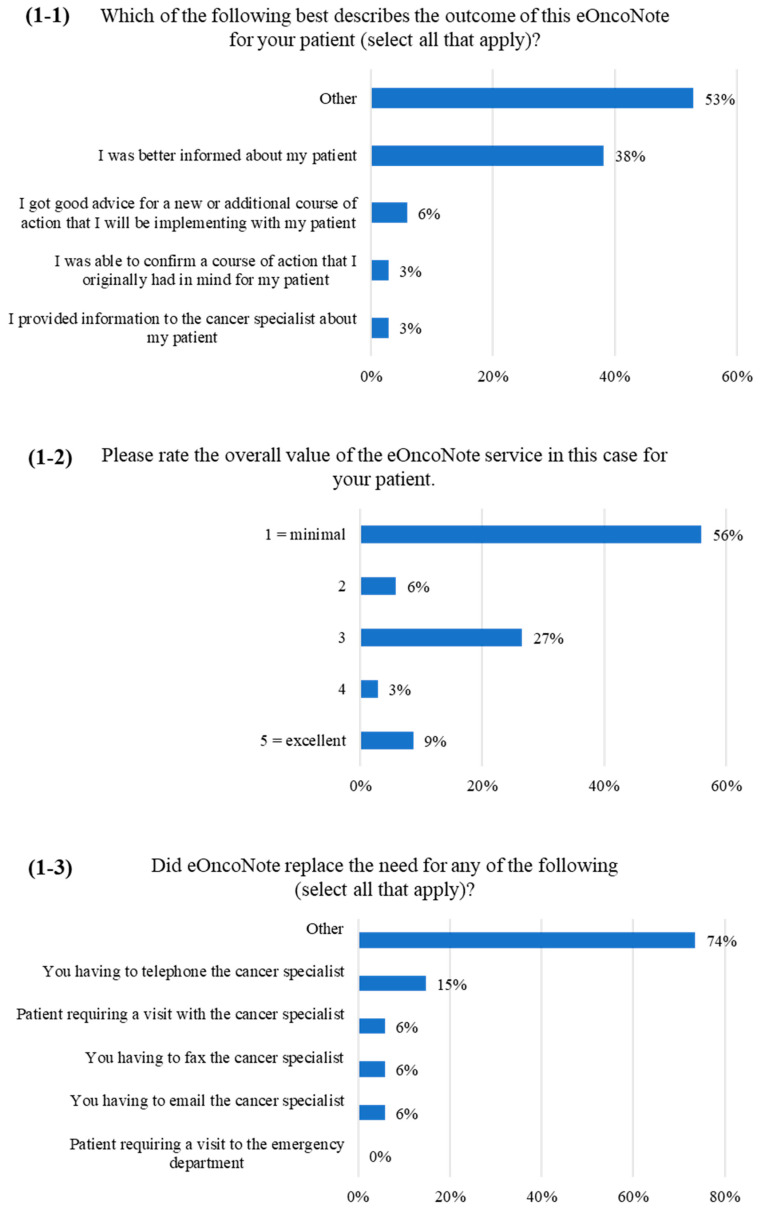
PCP survey responses ((**1-1**–**1-3**) correspond to items 1, 2 and 3 on the survey, respectively).

**Figure 2:**
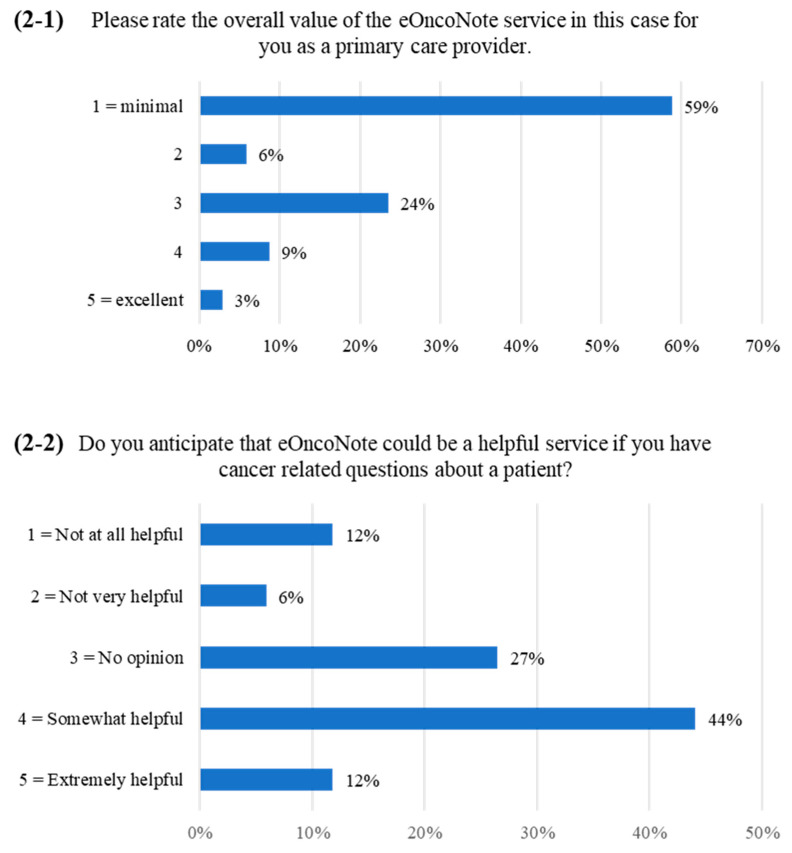
PCP survey responses ((**2-1**,**2-2**) correspond to items 4 and 5 on the survey, respectively).

Figure 1 and Figure 2 show the PCP survey results. When asked about the outcome of the eOncoNote discussion (Figure 1-1), 18 PCPs (53%) responded “other”, and 17 PCPs provided a response to specify for “other”. Seventy-one percent (12/17) commented that there was no benefit or the same benefit as having a faxed consult note (e.g., “information was redundant; already had a consult on file from this physician”), 18% (3/17) commented that there was a need for EMR integration, and 12% (2/17) entered other comments.

Over half of the PCPs (19/34, 56%) rated the overall value of the eOncoNote service for their patient as minimal (Figure 1-2). When asked about whether eOncoNote replaced the need for any specific action on the PCP’s part (Figure 1-3), most PCPs selected “other”. Of the 24 PCPs that provided a response to specify for “other”, 83% (20/24) commented that eOncoNote did not replace any other actions, while 17% (4/24) entered other comments (e.g., “it might have prevented me having to fax the cancer specialist if I had a question”). Over half of the PCPs (20/34, 59%) rated the overall value of the eOncoNote service for their particular patient case as minimal (Figure 2-1). However, when they were asked about whether they anticipate that eOncoNote could be a helpful service if they had cancer-related questions about a patient, over half of the PCPs (19/34, 56%) indicated that eOncoNote could be a “somewhat helpful” or “extremely helpful” service (Figure 2-2).

Thirteen PCPs (13/34, 38%) answered an optional open-ended question at the end of the survey to provide additional feedback. Five PCPs (5/13, 38%) provided examples of contexts in which eOncoNote could be useful (e.g., “this single case did not require much back and forth communication so may not demonstrate the value the system may have for a more complex patient that primary care has a more active role with”). Five PCPs (5/13, 38%) highlighted the need for EMR integration (e.g., “useful service but tedious to having to log in every time; it would be better if this service was integrated with EMR”). Three PCPs (3/13, 23%) provided other comments (e.g., “clinic notes from [hospital] are arriving within 48 h in most cases; is this service intended to replace the clinic note or duplicate it?”).

## 4. Discussion

We examined the implementation of a communication system for PCPs and cancer specialists by analyzing usage data and responses from a PCP survey. The results demonstrated that there was limited engagement from PCPs in eOncoNote discussions with cancer specialists. For most of the discussions, PCPs needed to be prompted to respond to the cancer specialist and complete the survey to close the case. Upon being reminded up to three times to respond to the initial eOncoNote message from the cancer specialist, about 40% of PCPs in each phase responded via eOncoNote. Some PCPs responded to the RA’s reminders via email, perhaps because they indicated in the survey that logging into a separate system was a barrier to use. PCPs have reported that being able to directly connect via email facilitated communication between PCPs and cancer specialists [20]. In both phases, most PCPs in this study sent only one message, mainly acknowledging that they received the information and thanking the cancer specialist. In a few of the case discussions in the treatment phase, the PCP and cancer specialist discussed the patient’s treatment and mental health concerns. Likewise, there were not many messages sent from the cancer specialists, as in most cases they only sent an initial invitation to communicate and then a final message to initiate case closure. This communication pattern emphasizes the interdependence of system adoption because of the interactive nature of communication technology, as outlined by the diffusion of innovations theory [21]. Since eOncoNote is an interactive innovation, PCPs and cancer specialists using the system have reciprocal interdependence, making the system increasingly valuable as more healthcare providers register and use the service. Similarly, if a PCP or cancer specialist does not respond to their colleague, the other clinician may be less likely to use the system or stop using it altogether.

The infrequent use of eOncoNote may be partly related to the pRCT design, since each PCP could only have one patient enrolled. Having the ability to discuss the care of more patients on the eOncoNote system may have improved the system’s adoption among PCPs [22]. It is also possible that there may have been less of a need for communication between PCPs and cancer specialists for this particular group of patients. In our qualitative interviews, healthcare providers noted that communication was generally one-directional with cancer specialists sending reports to PCPs by fax. Healthcare providers reported that PCPs were more likely to contact cancer specialists by telephone or fax after the patient completed treatment, in instances where a patient had more complex needs, or if the patient lived far away from the cancer center [18]. Results from the pRCT demonstrated that patients randomized to the intervention group had an average of three comorbid conditions in the treatment phase, and an average of two comorbid conditions in the survivorship phase [17], so there may have been less of a need for communication regarding patients who did not have as many illnesses.

During this study, PCPs were still able to communicate with cancer specialists via usual channels while the eOncoNote intervention was active. We abstracted hospital EMR data on PCP and cancer specialist communication via the usual channels, which indicated that there were few calls made by PCPs or faxes sent to the cancer center during the treatment and survivorship phases [17]. Taken together, these results indicate that PCPs were not communicating with cancer specialists via usual channels instead of using eOncoNote, rather there was limited communication beyond the faxed consult notes from the specialist. This suggests that in addition to facilitating communication through a web-based system, perhaps a co-intervention is also needed to move beyond the traditional one-directional consult notes being faxed from cancer specialists to PCPs. Educational outreach involving physician champions using case-based presentations with content specifically designed for target audience specialties may help increase the use of provider-to-provider communication tools [23]. Perhaps part of the resistance to adopting the eOncoNote system is related to the information overload that physicians experience through the use of EMRs [24], as well as frustration with having separate systems that are not integrated [8]. As such, there is a need for training about the optimal use of communication tools in medical school curricula [23], as well as oncology training for PCPs to support shared care [9,25].

In the survey, PCPs reported no additional benefits of eOncoNote beyond the standard faxed consult note they received from the cancer specialists. Most PCPs also indicated that eOncoNote did not replace any other actions they needed to take, and there was minimal benefit for their patients. However, over half of the PCPs indicated that eOncoNote could be a helpful service if they had questions about a patient. In response to two survey items, PCPs emphasized the need to integrate eOncoNote with their EMRs to avoid having to log into a separate system each time. This is consistent with data from our qualitative interviews [18], suggesting that EMR integration may be a major factor in successfully incorporating eOncoNote into the clinical workflow. Other studies have also reported similar barriers to adopting internet-based physician communication tools, including slow service, difficulty with accessing information and a time-consuming process [26].

The larger mixed methods study examined the effectiveness and implementation of the eOncoNote system through multiple perspectives. This sub-study provided insights about the usage of the eOncoNote system and added important contextual information that has helped to explain the findings from other study components, such as the qualitative interviews with healthcare providers. Survey responses helped to elucidate PCP perspectives on using eOncoNote, as few volunteered to participate in qualitative interviews.

A limitation of this study is that we did not reach our target patient recruitment in the treatment phase. PCPs could only have one patient participating in the pRCT (to reduce the potential for contamination between the intervention and control groups). Only PCPs who were already signed up for eConsult could participate, which reduced the pool of potential participants, and may have impacted the generalizability of the study results. With the extra challenges brought on by the COVID-19 pandemic, we decided to stop recruitment. Perhaps with additional patients in the treatment phase and patients with more complex health conditions, there may have been more eOncoNote messages exchanged between PCPs and cancer specialists. In addition, for patients in the survivorship phase, the discussions between PCPs and cancer specialists lasted for one year post-transfer from the cancer center to primary care. The one year period was selected for feasibility purposes, but it is possible that more questions may have arisen from PCPs if a longer interval had been chosen.

## 5. Conclusions

This study reported on the use of a communication system designed to facilitate the coordination of care between PCPs and cancer specialists. Our findings revealed limited use of the eOncoNote system and concerns with implementing it in primary care settings. Future studies should examine the implementation of communication systems alongside other interventions to support interprofessional collaboration between PCPs and cancer specialists, as well as opportunities for EMR integration.

## Figures and Tables

**Table 1:** Participant characteristics.

Characteristics	Treatment *n* = 33	Survivorship *n* = 43
Type of cancer specialist, *n* (%):		
Nurse	0	2 (100)
Oncologist	7 (100)	0
Type of primary care provider, *n* (%):		
Family physician	31 (94)	43 (100)
Nurse practitioner	2 (6)	0
PCP registration on eConsult, *n* (%):		
Previous	30 (91)	42 (98)
New	3 (9)	1 (2)
PCP reminders to respond to initial message, *n* (%):		
0 reminders	6 (18)	7 (16)
1 reminder	4 (12)	6 (14)
2 reminders	4 (12)	7 (16)
3 reminders	19 (58)	23 (53)
PCP responded to initial message, *n* (%)	13 (39)	17 (40)
PCP reminders to complete the survey, *n* (%):		
0 reminders	9 (27)	11 (26)
1 reminder	2 (6)	5 (12)
2 reminders	4 (12)	3 (7)
3 reminders	18 (55)	24 (56)
PCP completed survey, *n* (%):	12 (36)	22 (51)
Did not respond to initial message	3 (25)	10 (45)
Responded to initial message	9 (75)	12 (55)

**Table 2:** eOncoNote discussion data.

Characteristics	Treatment *n* = 33	Survivorship *n* = 43
Time from cancer specialist appointment to initial eOncoNote message being sent, *n* (%):		
0–4 days	12 (36)	12 (28)
5–9 days	8 (24)	20 (47)
10–14 days	2 (6)	7 (16)
15+ days	11 (33)	4 (9)
Time from RA notifying cancer specialist to send eOncoNote message to message being sent, *n* (%):		
0–4 days	27 (82)	38 (88)
5–9 days	3 (9)	2 (5)
10–14 days	1 (3)	0
15+ days	2 (6)	3 (7)
Time from sending eOncoNote to PCP response, *n* (%):		
0–4 days	4 (12)	5 (12)
5–9 days	1 (3)	1 (2)
10–14 days	1 (3)	2 (5)
15+ days	7 (21)	9 (21)
No response from PCP	20 (61)	26 (60)
Time from RA notifying cancer specialist to initiate case closure to message being sent, *n* (%):		
0–4 days	15 (45)	25 (58)
5–9 days	5 (15)	9 (21)
10–14 days	2 (6)	4 (9)
15+ days	7 (21)	3 (7)
missing	4 (12)	2 (5)
Time from cancer specialist initiating case closure to PCP survey completion, *n* (%):		
0–4 days	6 (18)	8 (19)
5–9 days	1 (3)	4 (9)
10–14 days	0	3 (7)
15+ days	5 (15)	7 (16)
Did not complete survey	21 (64)	21 (49)
PCP messages sent, *n* (%):		
0 messages	20 (61)	26 (60)
1 message	12 (36)	15 (35)
2 messages	1 (3)	2 (5)
Cancer specialist messages sent, *n* (%):		
1 message	1 (3)	2 (5)
2 messages	27 (82)	37 (86)
3 messages	4 (12)	3 (7)
4 messages	1 (3)	1 (2)
Content of eOncoNote discussions *, *n* (%):		
PCP confirmed receipt of information	3 (9)	4 (9)
PCP thanked cancer specialist	10 (30)	13 (30)
PCP and cancer specialist discussed patient’s treatment	2 (6)	0
PCP and cancer specialist discussed patient’s mental health	1 (3)	0
PCP indicated they are looking forward to the program	0	2 (5)
PCP asked whether there will be more communication	1 (3)	1 (2)
PCP informed cancer specialist about patient’s next appointment	0	1 (2)
PCP indicated they would prefer to use usual communication channels	0	1 (2)

* Content of eOncoNote discussions may include more than one category.

## Data Availability

Contact the corresponding author.

## References

[1] Doty M.M., Tikkanen R., Shah A., Schneider E.C. (2019). Primary care physicians’ role in coordinating medical and health-related social needs in eleven countries. Health Aff..

[2] Nielsen P., Sahay S. (2022). A critical review of the role of technology and context in digital health research. Digit. Health.

[3] Canadian Medical Association, Canada Health Infoway (2021). 2021 National Survey of Canadian Physicians. https://www.infoway-inforoute.ca/en/component/edocman/resources/reports/benefits-evaluation/3935-2021-national-survey-of-canadian-physicians?Itemid=101.

[4] Blumenthal D. The Electronic Health Record Problem. Commonwealth Fund. https://www.commonwealthfund.org/blog/2018/electronic-health-record-problem.

[5] Rudin R.S., Schneider E.C., Predmore Z., Gidengil C.A. (2016). Knowledge gaps inhibit health IT development for coordinating complex patients’ care. Am. J. Manag. Care.

[6] Winkfield K.M., Schlundt D.G. (2022). Creating the right team to ensure equitable cancer care: Whose job is it anyway?. JCO Oncol. Pract..

[7] Jones P., Shakdher S., Singh P. (2017). Synthesis maps: Visual knowledge translation for the CanIMPACT clinical system and patient cancer journeys. Curr. Oncol..

[8] Easley J., Miedema B., Carroll J.C., Manca D.P., O’Brien M.A., Webster F., Grunfeld E. (2016). Coordination of cancer care between family physicians and cancer specialists: Importance of communication. Can. Fam. Physician.

[9] Easley J., Miedema B., O’Brien M.A., Carroll J., Manca D., Webster F., Grunfeld E. (2017). The role of family physicians in cancer Care: Perspectives of primary and specialty care providers. Curr. Oncol..

[10] Easley J., Miedema B., Carroll J.C., O’Brien M.A., Manca D.P., Grunfeld E. (2016). Patients’ experiences with continuity of cancer care in Canada: Results from the CanIMPACT study. Can. Fam. Physician.

[11] Hahn E.E., Ganz P.A., Melisko M.E., Pierce J.P., von Friederichs-Fitzwater M., Lane K.T., Hiatt R.A. (2013). Provider perceptions and expectations of breast cancer posttreatment care: A University of California Athena Breast Health Network Project. J. Cancer Surviv..

[12] Grunfeld E., Petrovic B. (2017). Consultative workshop proceedings of the Canadian Team to Improve Community-Based Cancer Care Along the Continuum. Curr. Oncol..

[13] World Health Organization Classification of Digital Health Interventions v1.0. https://www.who.int/publications/i/item/WHO-RHR-18.06.

[14] Liddy C., Rowan M.S., Afkham A., Maranger J., Keely E. (2013). Building Access to Specialist Care through E-Consultation. Open Med..

[15] Champlain BASE eConsult Growth of a Sustainable Service. https://www.champlainbaseeconsult.com/growth-of-a-sustainable-service.

[16] Curran G.M., Bauer M., Mittman B., Pyne J.M., Stetler C. (2012). Effectiveness-implementation hybrid designs: Combining elements of clinical effectiveness and implementation research to enhance public health impact. Med. Care.

[17] Petrovic B., Julian J.A., Liddy C., Afkham A., McGee S.F., Morgan S.C., Segal R., Sussman J., Pond G.R., O’Brien M.A. (2023). Web-based asynchronous tool to facilitate communication between primary care providers and cancer specialists: Pragmatic randomized controlled trial. J. Med. Internet Res..

[18] Petrovic B., O’Brien M.A., Liddy C., Afkham A., McGee S.F., Morgan S.C., Segal R., Bender J.L., Sussman J., Urquhart R. (2022). Patient and healthcare provider perspectives on the implementation of a web-based clinical communication system for cancer: A qualitative study. Curr. Oncol..

[19] Hsieh H.-F., Shannon S.E. (2005). Three approaches to qualitative content analysis. Qual. Health Res..

[20] Dossett L.A., Hudson J.N., Morris A.M., Lee M.C., Roetzheim R.G., Fetters M.D., Quinn G.P. (2017). The primary care provider (PCP)-cancer specialist relationship: A systematic review and mixed-methods meta-synthesis. CA Cancer J. Clin..

[21] Rogers E.M. (2003). Diffusion of Innovations.

[22] O’Donnell A., Kaner E., Shaw C., Haighton C. (2018). Primary care physicians’ attitudes to the adoption of electronic medical records: A systematic review and evidence synthesis using the Clinical Adoption Framework. BMC Med. Inform. Decis. Mak..

[23] Walsh K.E., Secor J.L., Matsumura J.S., Schwarze M.L., Potter B.E., Newcomer P., Kim M.K., Bartels C.M. (2018). Secure provider-to-provider communication with electronic health record messaging: An educational outreach study. JHQ.

[24] Koopman R.J., Steege L.M.B., Moore J.L., Clarke M.A., Canfield S.M., Kim M.S., Belden J.L. (2015). Physician information needs and electronic health records (EHRs): Time to reengineer the clinic note. JABFM.

[25] Lisy K., Kent J., Piper A., Jefford M. (2021). Facilitators and barriers to shared primary and specialist cancer care: A systematic review. Support. Care Cancer.

[26] Barr N.G., Randall G.E., Archer N.P., Musson D.M. (2017). Physician communication via internet-enabled technology: A systematic review. J. Health Inform..

